# Frequency dependence of surface acoustic wave swimming

**DOI:** 10.1098/rsif.2019.0113

**Published:** 2019-06-19

**Authors:** C. Pouya, K. Hoggard, S. H. Gossage, H. R. Peter, T. Poole, G. R. Nash

**Affiliations:** 1 College of Engineering, Mathematics and Physical Sciences, University of Exeter, Exeter EX4 4QF, UK; 2 Natural Sciences, University of Exeter, Exeter, EX4 4QF, UK

**Keywords:** surface acoustic waves, microfluidics, microswimmers, acoustic streaming, laminar jets

## Abstract

Surface acoustic waves (SAWs) are elastic waves that can be excited directly on the surface of piezoelectric crystals using a transducer, leading to their exploitation for numerous technological applications, including for example microfluidics. Recently, the concept of SAW streaming, which underpins SAW microfluidics, was extended to make the first experimental demonstration of ‘SAW swimming’, where instead of moving water droplets on the surface of a device, SAWs are used as a propulsion mechanism. Using theoretical analysis and experiments, we show that the SAW swimming force can be controlled directly by changing the SAW frequency, due to attenuation and changing force distributions within each SAW streaming jet. Additionally, an optimum frequency exists which generates a maximum SAW swimming force. The SAW frequency can therefore be used to control the efficiency and forward force of these SAW swimming devices. The SAW swimming propulsion mechanism also mimics that used by many microorganisms, where propulsion is produced by a cyclic distortion of the body shape. This improved understanding of SAW swimming provides a test-bed for exploring the science of microorganism swimming, and could bring new insight to the evolutionary significance for the length and beating frequency of swimming microbial flagella.

## 1. Introduction

The properties of surface acoustic waves (SAWs) have been investigated since Lord Rayleigh delivered the first mathematical discussion on the propagation of waves on the free surface of an elastic solid in an address to the London Mathematical Society in 1855 [1]. However, it was the invention of the interdigital transducer (IDT) in 1965 by White & Voltmer [2], allowing SAWs to be directly excited on the surface of piezoelectric crystals, that enabled SAW devices to be developed for applications such as signal processing. Over the last decade, or so, SAW devices have also had a growing presence in the field of microfluidics [3,4] due to the phenomenon of acoustic streaming [5], where SAWs can be used to induce fluid motion. When a water droplet is applied to the surface of a SAW device, the propagating SAWs are converted into ‘leaky SAWs’, which are radiated into the liquid and decay, causing fluid motion [5,6]. This effect can cause droplet formation, vibration and movement as well as ejection of smaller droplets [6,7]. Applications of acoustic streaming include micro-manipulators for small particles or cells [8], microchannel transport [9], atomization [10,11], microfluidic-mixing [12], among many others [13].

Very recently, Bourquin & Cooper [14] extended the concept of SAW streaming [6] to present the first, and only reported, experimental demonstration of ‘SAW swimming’, where instead of moving droplets on the surface of a device, SAWs are used as an aquatic propulsion mechanism for a centimeter scale vessel. In this work, we report the first theoretical model of this phenomenon, and use this model to investigate an overlooked property of SAW swimming: the frequency dependence of the swimming force. We show that there is an optimum frequency which generates the maximum force, which we confirm in an experimental study.

Ultimately, this improved understanding could lead to the development of artificial swimming devices, with no moving parts, for applications such as minimally invasive endoscopic surgery. In addition, these results might also help further the understanding of the fundamental science of microorganism swimming, who use similar cyclic movements to propel themselves.

## 2. Results

### 2.1. Theoretical analysis and discussion

**Figure 1.**
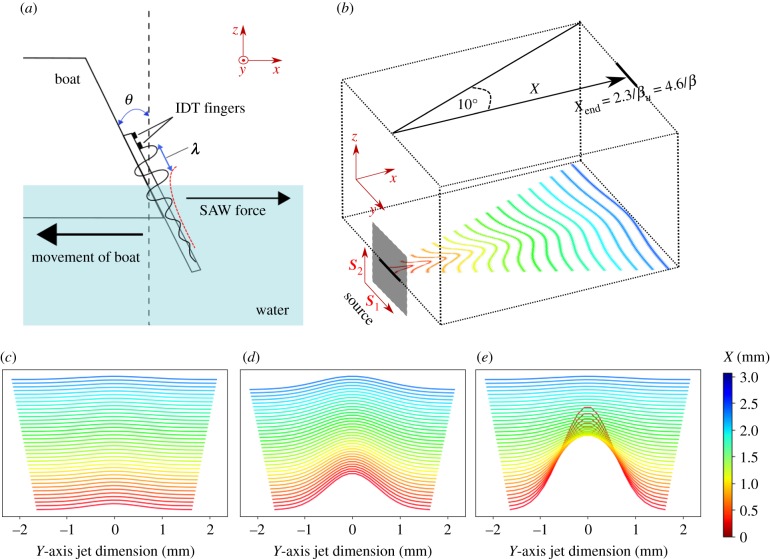
SAW swimming device and jet arrangement. (*a*) Schematic of the boat design and SAW swimming phenomenon, showing the SAW exponentially decaying (red dotted line) as it hits the water and turns into a leaky SAW, producing a SAW streaming force from the rear of the boat and driving the vessel forwards. λ is the SAW wavelength = 2***d***, where ***d*** is determined by the separation between fingers of the IDT. The fundamental frequency of the IDT is 11 MHz. (*b*) Schematic of the jet emitted from the SAW source with Gaussian beam divergence and edge of jet definitions from Dentry *et al*. [16]. Also shown are axes of the source dimension coordinates $s_{1}$ and $s_{2}$ (relating to $L_{1}$ and $L_{2}$). (*c–e*) Off-axial profiles are shown as a set of Gaussians scaled by an axial force profile taken from the main image in figure 2*b*. Gaussian force profiles across the jet close to the source (less than 3 mm) are shown for clarity (*c*) below the peak frequency (11 MHz), (*d*) around the peak frequency (56 MHz) and (*e*) above the peak frequency (146 MHz). (Online version in colour.)

The frequency dependence of the SAW streaming force was identified by performing an in-depth study of the leaky SAW jet profile in di-ionized (DI) water. The theoretical physics describing the velocity produced by SAW streaming was discussed in-depth by Lighthill [15] with alterations to this theory in a recent paper by Dentry *et al*. [16] which thoroughly analysed the axial jet velocity profile of a SAW streaming jet produced from a configuration similar to the SAW swimming system presented here. Dentry *et al*. fixed a SAW device in place and the SAW device was positioned at an angle of *θ* = 0°, so that the SAW streaming force was not directed parallel to the surface of the water. In our study, the device is free to move and is positioned at the Rayleigh angle of 23° [14], as shown schematically in figure 1*a*, to allow the vessel to produce a maximum force in the direction of propulsion. The Rayleigh angle is given by

2.1 $$\theta_{R}=\sin^{-1}\frac{v_{F}}{v_{\mathrm{SAW}}}\;.$$

**Figure 2.**
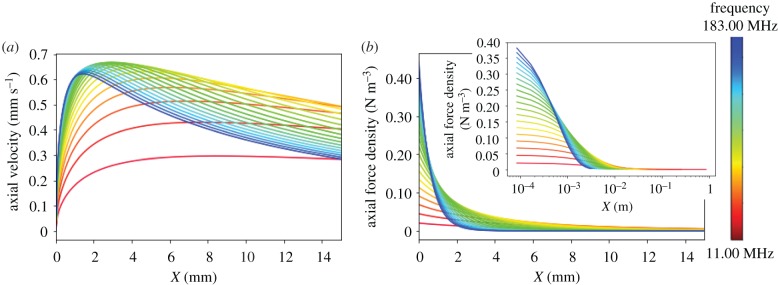
Calculated velocity and force profiles. (*a*) Axial velocity profile (across *X*) within the jet calculated by equation (2.2) with 20 different SAW input frequencies close to the source (less than 15 mm) measured in linear steps from 11 to 183 MHz. (*b*) Axial force per unit volume profile close to the source (less than 15 mm) calculated by equation (2.3) for 20 different SAW input frequencies and (inset) until the edge of the jet where the power reaches 1% of the initial value. (Online version in colour.)

Here $v_{F}$ is the velocity of the longitudinal wave in the fluid, and $v_{\mathrm{SAW}}$ is the velocity of the SAW.

Although Dentry *et al*. perform an in-depth analysis of velocity profiles within SAW streaming jets, Lighthill [15] and Dentry *et al*. [16] also briefly discuss the equations governing SAW streaming forces within the jet. We extend the analysis performed by Lighthill [15] and Dentry *et al*. [16] to analyse force distributions within SAW streaming jets and from this we determine the theoretical thrust of a SAW swimming device. The jet emanates from the source and follows an axial jet trajectory along *X* as shown in figure 1*b*. Perpendicular to this, the jet's velocity and force profiles are assumed to follow a Gaussian distribution and undergo spreading, emanating from the source. The purely axial velocity and force density profiles for each frequency are described as follows [16]:

2.2 $$u(X,s_{1},s_{2})=\sqrt{\frac{2f(X)}{\pi S_{1}(X)S_{2}(X)}}$$

and

2.3 $$F(X,s_{1},s_{2})=\frac{\rho {\;f}^{\prime }(X)}{\pi K_{1}(X)K_{2}(X)},$$

where *u* is the jet velocity and *S* is related to the beam spread in the dimension of the reference frame of the source, perpendicular to *X*. $\;s_{1}$ and $s_{2}$ are the axes of the source. *S* and *K* describe the spread of the jet at a position *X* across the length of the jet. For our study, we assume $S_{i}(X)$ and $K_{i}(X)$ to be equal to the radius of an almost conical jet at each point along *X* in the coordinates perpendicular to the axial jet profile. *S* at each point along *X* is determined by the finite source width (the IDT aperture *L*_1_ = 3.25 mm) and the beam spread with semiangle of 10° [16]. The length of the device submerged in the water was taken as the same length *L*_y_ = 3.25 mm, for all frequencies representing a typical submerged length during SAW swimming experiments (total length of an experimental device = 5.4 mm). Therefore, the projection of this at 23° [16] was taken as the source dimension in the second dimension *L*_2_ to account for the Rayleigh angle. Finite source dimensions and beam spread were included due to the analysis performed by Dentry *et al*. [16], who verified experimentally that the inclusion of a finite source dimension to Lighthill's model [15] allowed accurate theoretical analysis to be made of velocity profiles within the jet. Dentry *et al*. also showed that a beam spread with semiangle of approximately 10° was consistent for all analysed frequencies from 19.7 to 936 MHz over a range of powers. A schematic of this is shown in figure 1*b*. *F* is the acoustic body force per unit volume and ρ is the density of the liquid (998 kgm^−3^). f(X) is related to the total momentum flux across the jet cross-section at constant *X* and is described by

2.4 $$f\;(X)=\frac{1}{\rho c}P[1-e^{-\beta X}],$$

where *c* is the speed of sound in the liquid (1498 ms^−1^), β is the attenuation coefficient of the beam power $\beta =2\beta_{u}=(4/3)(\mu +\mu^{\prime })\omega^{2}/\rho c^{3}$ [16], where μ is the shear viscosity of the fluid (10^−3^ Pa s^−1^), $\mu^{\prime }$ represents the bulk viscosity of the fluid (taken as 2.47 × 10^−3^ Pa s, [17]) and ω is angular frequency. Therefore, the attenuation coefficient of the beam power β is frequency-dependent. ${\;f}^{\prime }(X)$ is the spatial derivative of f(X). *P* is the total power of the sound beam, which is related to the power at the source, and decays along the substrate when the SAW is in contact with the fluid due to attenuation [16]. When in the water, the power along the substrate decays as $e^{-2\alpha y}$ [16], where *α* is the SAW attenuation $\mathrm{coefficient}=\rho c/\rho_{s}V_{\mathrm{SAW}}\lambda_{\mathrm{SAW}}$, $\rho_{s}$ is the density of the substrate and $\lambda_{\mathrm{SAW}}$ is the SAW wavelength. The total power was considered as the integral of this power decay [16] across the device from 0 to *L_y_* (the submerged second source dimension) multiplied by an original power *P*_0_ before the SAW is in contact with the fluid (taken as an estimate of *P*_0_ = 25 mW as a typical input power expected from experiments) (i.e. $P=P_{0}\int_{0}^{Ly}e^{-2\alpha y}dy$ where *y* is the distance travelled along the substrate which has been adapted from the power decay equation $P=\int_{0}^{\infty }\rho wc{\dot{\eta }}_{m}^{2}e^{-2\alpha y}dy$ from Dentry *et al*. [16], where *w* is the width of the wavefront and ${\dot{\eta }}_{m}$ is the vibrational velocity before contact with a fluid). The SAW attenuation coefficient *α* describes the wavelength-dependent, and therefore frequency-dependent, attenuation across the substrate, the second frequency-dependent attenuation in the system. These variables are all constituent variables in the velocity equation (equation (2.2)) and force equation (equation (2.3)). In turn, this results in a highly frequency-dependent velocity equation (equation (2.2)) and force equation (equation (2.3)). Perpendicular to the axial direction, the jet's velocity and force profiles exhibit a Gaussian distribution, with the maximum velocity or force positioned at the axial position within the jet. As such, the force and velocity profiles across the length at off-axis points across the jet width have a similar form but with reduced absolute values as given by Dentry *et al*. [16] (i.e. $u(X,s_{1},s_{2})=\sqrt{\left(2f\;(X)/\pi S_{1}(X)S_{2}(X)\right)}\exp[-{\left(s_{1}/S_{1}\right)}^{2}-{\left(s_{2}/S_{2}\right)}^{2}]$ and $F(X,s_{1},s_{2})=(\rho {\;f}^{\prime }(X)/\pi K_{1}(X)K_{2}(X))\exp[-{\left(s_{1}/K_{1}\right)}^{2}-{\left(s_{2}/K_{2}\right)}^{2}]$). Here, to simplify the model, we consider only axial velocities and forces.

The axial velocity profile of jets with SAW frequencies in an experimentally accessible range were calculated using equation (2.2) and are plotted in figure 2*a*. The distributions are shown clearly at positions within the jet close to the source, as shown in figure 2*a,b* where velocity profiles are plotted up to a maximum of 15 mm. Figure 2*a* shows that the peak velocity increases and its position moves closer to the source with increasing frequency, consistent with previous studies [16]. The beam length, considered by Dentry *et al*. [16] to end when the power is reduced to 1% of the initial power is reduced to a smaller length with increasing frequency (figure 2*b* inset and electronic supplementary material, table S1). The end of the beam, i.e. position within the jet where the power is reduced to 1% of the power at the source, *P*, can be shown to be located at $X_{\mathrm{end}}=2.3/\beta_{u}=4.6/\beta$ and therefore proportional to $1/\omega^{2}$ (figure 1*b* and electronic supplementary material, table S1) [16]. The peak velocity is presented in electronic supplementary material, figure S1 and the differences between our study and Dentry *et al.* is likely to arise due to the differences in our models including the different values of *L*_1_, *L*_2_ and *L_y_* used. Previous studies [16,18] have analysed the peak velocity for different frequency jets, yet none have analysed the frequency dependence on force.

**Figure 3.**
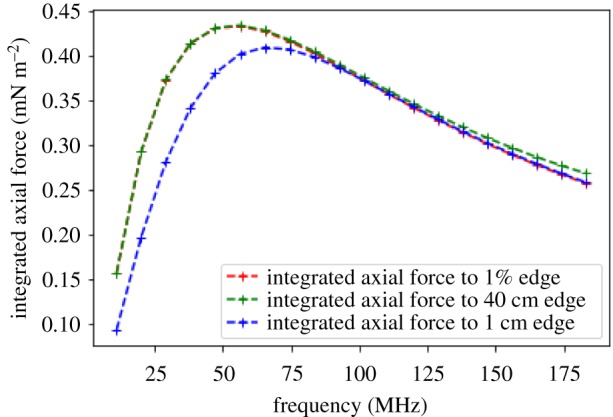
The axial force calculated from equation (2.3), and integrated across *X* up to the edge of the jet for 20 different SAW input frequencies. The edge of the jet was considered to be when the jet power reaches 1% of the source power with attenuation length dependence varying as $1/\omega^{2}$ (red curve), we also consider two standard jet lengths within the confines of a finite container with standard length of 40 cm (green curve) and 1 cm (blue curve) for all frequencies. The red curve tends to that of the green curve at lower frequencies (with lower frequency peaks) and to the blue curve at higher frequencies (with a slightly lower integrated axial force). (Online version in colour.)

The axial force density of 20 different jets with SAW frequencies within an experimentally accessible frequency range were calculated from equation (2.3) (figure 2*b*). Figure 2*b* shows the force distributions of all frequency jets within an axial distance close to the source (less than 15 mm) and the inset shows the whole length of each profile until the end of the jet, where the power reaches 1% of the power at the source. Each force profile, for each frequency, was integrated over the distance *X* from the source until its associated 1% jet edge, using the Python numpy.trapz package function. The results are plotted in figure 3, which show that there is a maximum integrated axial force at a SAW frequency of approximately 56 MHz. Additionally, we also adapt the theoretical model to consider total jet lengths that are not limited to above 1% of the initial power. Figure 3 also shows the integrated axial forces calculated using two standard jet lengths for all frequencies within the confines of a container, one with standard length of 40 cm (the maximum length of the water container used in the experiments and presented in a later section) and one smaller size of 1 cm to represent a theoretical minimum possible jet length within a container. The differences in the force profiles for jet lengths considered within a finite domain and up to the 1% jet edge are minimal (figure 3). For the 1 cm jets, only the lower frequency axial forces decrease slightly when compared with the 1% power jets. For the 40 cm jets, there is a small increase in the axial force at higher frequencies where highly attenuated forces from beyond the 1% jet edge are now included in the jet calculations. A slight difference in the absolute axial force values exist at lower and higher frequencies when considering constant finite jet lengths for all frequencies, but a peak force is still present with a slight shift in its frequency.

The physical origin of the peak force can be understood by interpretation of the force profiles in figure 2*b* and by analysing the redistribution of forces within each jet with respect to the change in SAW input frequency. The key to changing forces within each jet arises from two frequency-dependent attenuations; the attenuation of the jet within the water (with *β*-dependence) and the atenuation of the power along the substrate (with *α*-dependence). Analysis of force profiles show that lower SAW input frequencies produce a steadier small axial force with gradual changes throughout the long jet length. As the SAW input frequency is increased, the axial force close to the source increases. Additionally, with increasing frequency, attenuation greatly reduces the length of the jet comprising substantial forces and so forces close to the source contribute significantly to the total integrated axial force. The maximum force close to the source increases linearly with frequency, while the attenuation of jet length is proportional to $1/\omega^{2}$, However, each frequency has its own force expression which incorporates these factors, and is a combination of *α* (frequency-dependent SAW attenuation coefficient), β (the frequency-dependent attenuation coefficient of the beam power) and exponentials of these terms (all of which are frequency-dependent) which produces a unique curve shape/force profile for each frequency jet. There exists an optimum frequency, where the initial axial force is high and the force profile is such that larger forces are maintained for longer distance, but attenuation of the jet is not considerable, resulting in larger axial forces over longer distances. Beyond this frequency, attenuation of the jet reduces the jet lengths comprising substantial forces and so the total integrated axial force at higher frequencies is significantly reduced. When changing frequency, the balance of increasing forces close to the source and the changing force distributions due to the two frequency-dependent attenuations of the jet give rise to a changing total integrated axial force and a peak in the integrated axial force at around 56 MHz.

As non-axial forces are represented by the axial force scaled by a Gaussian, a schematic representation of the axial force close to the source (less than 3 mm) within the jet of a frequency below the peak frequency (11 MHz), around the peak frequency (56 MHz) and above the peak frequency (146 MHz) are shown in figure 1*c,d* and *e*, respectively. It is apparent from the Gaussian representation that attenuation produces higher forces (peaks in figure 1*c–e*) close to the source, over shorter distances when frequency is increased, but attenuation means these forces are only present over short distances. Comparatively, the lower frequency representation (figure 1*c*) shows low forces and the optimum frequency (figure 1*d*) has consistently larger forces over longer distances.

### 2.2. Experimental results and discussion

To validate the experimental set-up, which consisted of a SAW device mounted on a polystyrene vessel, the net forward force resulting from the SAW force was first measured as a function of SAW power at a SAW frequency of 11 MHz, as shown in electronic supplementary material, figure S2. The measured swimming force increases approximately linearly with increasing SAW power, in agreement with theory (equations (2.3) and (2.4)) and previous experiments [14]. The size of the measured SAW swimming force is also consistent with the maximum value, 8 mN, obtained by Bourquin & Cooper [14] at a SAW frequency of approximately 11 MHz, transducer aperture of 15 mm, and an acoustic power of 1.7 W. In our case, we obtain a value of the SAW swimming force 0.04 mN at 11 MHz, but for a transducer aperture of 3.25 mm, and an estimated acoustic power of approximately 25 mW (taking into account the transmission coefficient of the IDTs and other losses in the system). Correcting for these factors, would give a maximum SAW swimming force of 12 mN.

**Figure 4.**
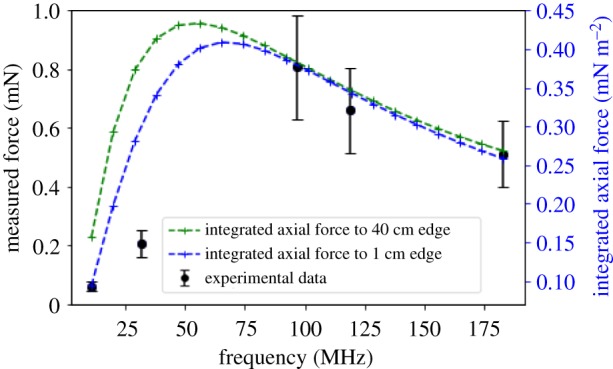
The experimental SAW forces measured over the time period of 0.5 s at five resonant input frequencies of the SAW device (11, 32, 97, 119 and 183 MHz), normalized by systematic errors from the experimental set-up including the transmission coefficient of the IDTs. Also shown are the theoretical integrated axial force calculated from equation (2.3) and integrated across *X* up to a jet length of 40 cm and 1 cm for 20 different SAW input frequencies spanning the frequency range used in the experiment. Error bars calculated from combined error equation. (Online version in colour.)

To investigate the frequency dependence of the SAW swimming force, a second SAW device was mounted onto a boat of the same design. In this case, the RF signal was pulsed (period of approx. 1.5 s) to prevent excessive heating of the SAW device [14]. The acoustic power was approximately 25 mW. The movement of the boat was then measured at five different input frequencies: 11, 32, 97, 119 and 183 MHz and the net forward forces resulting from SAW forces were calculated at 0.5 s after the application of the RF signal, at each frequency. The measured force, corrected for the frequency response of the system and normalized to the fundamental frequency response of the IDT, is plotted as function of frequency in figure 4 and shows a strong nonlinear dependence on the SAW frequency (note that due to the discrete resonances of the IDTs, values of the force could not be obtained at frequencies between 32 MHz and 97 MHz). For comparison, values of the swimming force calculated from theory are also plotted in figure 4. Overall, there is good agreement between the frequency dependence of the measured and calculated forces. The difference in the measured and calculated values of the force, with the calculated forces approximately half of the measured forces, is because only axial forces were considered in the theoretical calculation. As the jet spreads away from the axial line, the force distributions are reported to be the same [15,16] with smaller absolute values, represented by a Gaussian distribution. Therefore, it is practical that the theoretical force distribution at off-axial angles will simply increase in absolute values when these off-axial forces are also considered.

Additionally, the theory assumes that the SAW source is static, whereas in the experiments the vessel is moving. This is likely to produce a more complicated beam profile, due to interference, and could change the properties of the SAW swimming efficiency. Movement may also slightly alter the length of the submerged region of the device, giving rise to slightly changing input powers due to the changing source dimension *L_y_*, which could alter the force distribution slightly (see the electronic supplementary material, figure S3). Further work is therefore underway to extend the theoretical analysis to include the effects of a moving vessel, and to undertake new experiments using SAW devices designed specifically for SAW swimming. It should be noted that the frequency at which there is a peak swimming force is likely to vary between different experimental configurations. Finally, the use of a substrate with a slower SAW velocity, such as glass, would allow the development of swimming devices that produce the maximum thrust when they lie almost parallel to the surface of the fluid. This will reduce their macroscopic drag, but will also remove the requirement for the SAW device to be mounted on a centimetre size vessel. In this case, the SAWs could also be excited remotely using a laser [19].

## 3. Conclusion

We theoretically predict and experimentally observe the presence of an optimum frequency for a SAW swimming device which gives rise to a maximum net forward force or thrust when placed in water. The value of the optimum frequency is reliant on the frequency-dependent attenuating properties of the surrounding fluid. For our experimental study, the optimum frequency lies between 32 and 97 MHz, but it should be noted that this frequency is likely to vary between different experimental configurations. Our experimental study is consistent with the results we obtained from a theoretical model which shows that the peak force for this device arises at a frequency of approximately 56 MHz, and that the force profiles within the jet have very different distributions at different frequencies. The main differences result from attenuations of the jet which consequently change the absolute values of axial force across the jet which dramatically alters the total integrated axial forces. This improved understanding, at a fundamental level, of this phenomenon, provides a foundation on which to design devices with improved performance and functionality. It could underpin future work to develop swimming devices with no moving parts, for applications such as minimally invasive endoscopic surgery. In addition, the use of multiple SAW frequencies, with different induced forces, could allow precise control of fluids in lab-on-a-chip applications, including SAW sorters [20]. Recent studies [21,22] have altered IDT geometries and substrates in order to add additional frequency control to their SAW streaming systems, additional alterations to the system could allow additional frequency control to a SAW streaming system.

Finally, it is interesting to note that the phenomenon of SAW swimming mirrors the propulsion mechanism of many microorganisms, such as bacteria, who move to find food, shelter and escape predators. Microorganisms have evolved methods of movement to overcome and exploit drag, in their low Reynolds number environment, where propulsion is produced by a cyclic distortion of their body shape [23–25], with similar wavelengths to that of the SAWs used here [23–27]. Some hypothesize that SAW motion and acoustic streaming is a likely propulsion method for some bacteria [28]. Different swimming strategies include using cilia and flagella, or by small multicellular organisms [25]. In many cases, the technological design of medical micro-robotics are inspired by, or similar to, swimming microorganisms [29]. For instance, the spiral micro-robot designed by Ishiyama *et al*. [30], which is similar to some cilium propulsion mechanisms, or the artificial bacteria flagella [31,32] which use magnetics to induce motion. In addition to magnetic propulsion, a recent review [29] highlighted other methods of medical micro-robotic propulsion including ‘propulsion by bubbles', ‘propulsion by chemical reaction’ and ‘propulsion by biological mechanism’ as methods of propulsion for medical swimmers. The experimental investigation of micro-swimming is challenging due to the difficulty in controlling the relevant key parameters, such as the wavelength of the cyclic distortion. Using SAWs, we have shown that there is a frequency/wavelength dependence of the force produced by a similar cyclic motion, which if it exists for flagella locomotion could pose an evolutionary significance relating the beating frequency, wavelength or length of flagella to producing an optimum or more efficient force. If so, an optimum beating frequency, or length, of the flagella of individual swimmers could also be tailored to their native fluid environment, which might possess specific attenuating or viscosity properties. SAW swimming devices can therefore be used as a test-bed to allow greater insight into the science underlying microorganism movement.

## 4. Material and methods

To test these results, the frequency response of a SAW swimming device was measured experimentally. A commercially available 128° YX LiNbO3 SAW delay line, with a centre-to-centre IDT separation distance of 5.4 mm and IDT aperture of 3.25 mm, was mounted on a printed circuit board (PCB) using conductive silver epoxy and 25 µm diameter bond wires. The IDTs had a double-digit geometry, allowing the efficient excitation of SAWs at a number of discrete resonant frequencies. The SAW wavelength *λ*_SAW_ = 2*d*, where *d* is determined by the separation between fingers of the IDT. The fundamental frequency of the IDT is 11 MHz. To form the swimming device, the PCB was mounted onto the rear of a polystyrene vessel using non-porous adhesive carbon tape, dimensions 40 mm × 25 mm × 30 mm (length, height and width, respectively) and mass 2.3460 ± 0.0005 g (figure 1*a*). The swimming device was placed in a container, dimensions 0.4 m × 0.4 m × 0.1 m, filled with several centimetres of DI water and free to move while under test. The rear of the boat was sculpted at an angle of 23° to the vertical axis to allow the SAWs to refract into the water and to propagate parallel to the surface of the water [8], as defined by the Rayleigh angle, given by equation (2.1). The device was positioned to account for the Rayleigh angle of the refracted SAW, so that the SAW streaming force was directed parallel to the surface of the water, resulting in the vessel being propelled in the opposite direction. Movement of the vessel was induced by exciting SAWs at the input transducer of the device using a connected Hewlett–Packard 8648C RF signal generator, the output from which was amplified using a Mini-circuits TVA-R5-13A amplifier. The displacement of the vessel over a defined period of time (0.5 s), from the application of the RF signal, was recorded using a GoPro Hero4 Silver camera at a rate of 60 frames per second. The time interval of 0.5 s was chosen as it was found to minimize the effects of the drag and resistive forces, arising from the surrounding water and the restoring force of the connecting wires, on the measurement of the force.

From the recorded data, the acceleration of the vessel was calculated using the equations of motion ($a=2s/t^{2}$, where *a* is the acceleration of the vessel and *s* is the distance travelled in time *t*) and the net forward force resulting from the SAW force was calculated using Newton's second law. This is the resultant forward force from the SAW force, reduced by drag forces from the surrounding water and the mechanical resistance experienced due to tethering from the connecting cables.

## Supplementary Material

Supplementary Information

## Supplementary Material

Data Accessibility

## Data accessibility

All data supporting this study are provided in the results section of the paper or as electronic supplementary material, accompanying this paper.
