# Supplementary material for: Frequency dependence of surface acoustic wave swimming
Source: J R Soc Interface. 2019 Jun 19;16(155):20190113. doi: 10.1098/rsif.2019.0113 (PMC6597766; doi:10.1098/rsif.2019.0113)
Supplement: Supplementary Information [file rsif20190113supp1.pdf]

Journal of the Royal Society Interface

Supplementary Information for

Frequency dependence of surface acoustic wave swimming

C. Pouya, K. Hoggard, S. H. Gossage, H. R. Peter, T. Poole and G. R. Nash

The supplementary material includes:

Figs. S1 to S3

Table S1

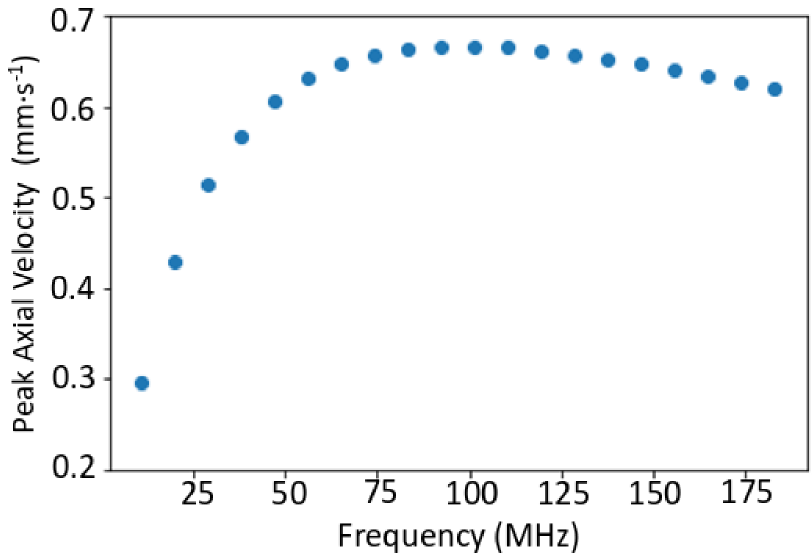

Fig. S1. The peak axial velocity found from velocity maxima in figure 2a.

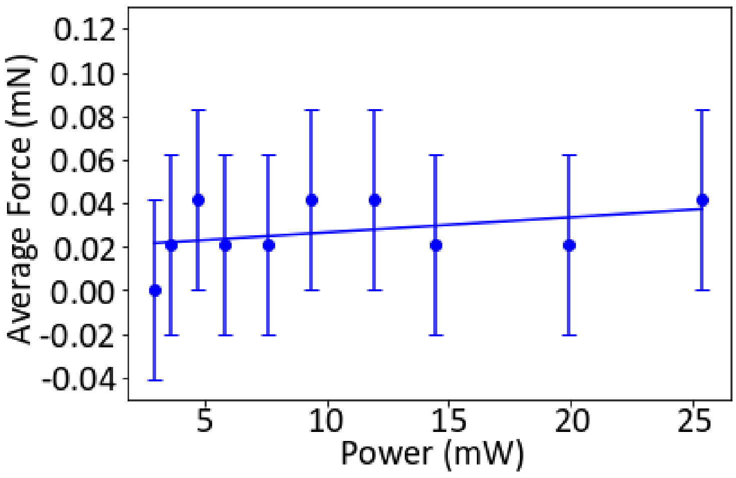

Fig. S2. The effect of input power on SAW force. Experimentally determined SAW force as a function of varying input power at 11 MHz over a period of 0.5s.

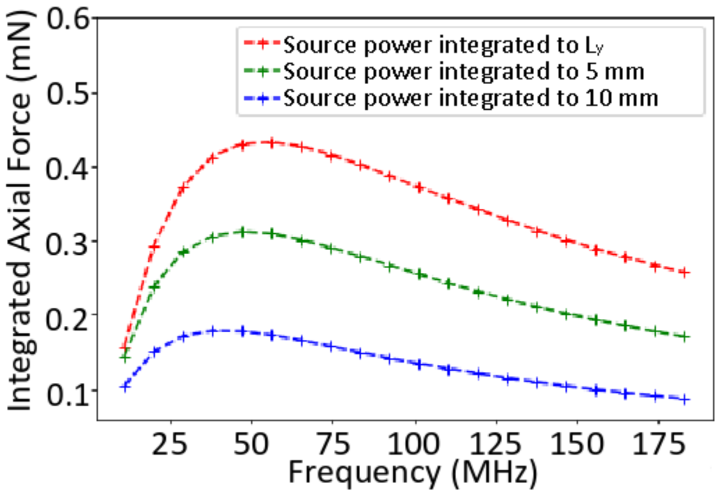

Fig. S3. Changing the source dimension and its effect on axial force. The source power has been integrated along the dimension y of the device, to L_y , the length of the submerged region of the device, as in the main text, and also to larger submerged lengths of 5 mm and 10 mm. The source dimension, L_2 , is then taken as this value projected to the 23° Rayleigh angle with the dimension L_1 remaining constant. The result of changing this depth is a change in the frequency producing the maximum axial force. Additionally the integrated axial force is reduced when larger source dimensions are considered due to the larger jet width resulting in power distribution over a larger volume within the jet, reducing the force density along the purely axial direction.

Table S1. Jet lengths until 1% power. Jet lengths (to 4 decimal places) calculated to where the power reaches 1% of the power at the source, P , after attenuation.

Frequency (MHz)	1% Jet Edge Length (m)
**11.00**	0.8493
**20.05**	0.2556
**29.10**	0.1213
**38.16**	0.0706
**47.21**	0.0461
**56.26**	0.0324
**65.32**	0.0241
**74.37**	0.0185
**83.42**	0.0147
**92.47**	0.0120
**101.53**	0.0099
**110.58**	0.0083
**119.63**	0.0071
**128.68**	0.0062
**137.74**	0.0054
**146.79**	0.0047
**155.84**	0.0042
**164.89**	0.0037
**173.95**	0.0033
**183.00**	0.0031
